# Serum EA-IgA and d-dimer, but not VCA-IgA, are associated with prognosis in patients with nasopharyngeal carcinoma: a meta-analysis

**DOI:** 10.1186/s12935-021-02035-2

**Published:** 2021-06-30

**Authors:** Tianhao Liang, Weixing Liu, Junyang Xie, Yiyan Wang, Gui Chen, Wenjing Liao, Lijuan Song, Xiaowen Zhang

**Affiliations:** State Key Laboratory of Respiratory Disease, Department of Otolaryngology-Head and Neck Surgery, First Affiliated Hospital, Guangzhou Medical University, #151 Yanjiang Road, Guangzhou, 510120 Guangdong People’s Republic of China

**Keywords:** EA-IgA, d-Dimer, VCA-IgA, Nasopharyngeal carcinoma, Prognosis, Meta-analysis

## Abstract

**Background:**

Patients with nasopharyngeal cancer (NPC) differ in prognosis, even at the same stage; therefore, new biomarkers are urgently required to identify early-stage NPC patients at high risk of poor prognosis. Although Epstein–Barr virus (EBV) DNA has been used for prognosis, the value of many other biomarkers expressed during the infection cycle of EBV remains unclarified. This study aimed to evaluate the prognostic potential of EA-IgA, VCA-IgA and d-dimer in patients with NPC.

**Methods:**

Electronic databases, including PubMed, Embase and Web of Science, were searched up to February 1, 2021. Pooled data were extracted from studies that evaluated the relationship between NPC and overall survival (OS), distant metastasis-free survival (DMFS) or disease-free survival (DFS) and then were subjected to a meta-analysis.

**Results:**

Nine studies with 5729 patients were included in this meta-analysis. In patients with NPC, EA-IgA levels significantly predicted OS (HR = 1.63, 95% CI 1.07–2.48). d-Dimer levels significantly predicted OS (HR = 1.75, 95% CI 1.24–2.47) and DMFS (HR = 1.91, 95% CI 1.31–2.79). However, high levels of VCA-IgA were not associated with OS (HR = 1.24, 95% CI 0.95–1.60), DMFS (HR = 1.41, 95% CI 0.92–2.17) or DFS (HR = 2.39, 95% CI 0.78–7.26).

**Conclusions:**

The present findings reveal that EA-IgA and d-dimer, but not VCA-IgA, can be used as prognostic biomarkers in NPC.

## Background

Nasopharyngeal carcinoma (NPC) is a common epithelial carcinoma arising from the nasopharyngeal mucosal lining. In comparison with other cancers, NPC is characterized by an unbalanced global geographic distribution and a high incidence in East and Southeast Asia [1].

Despite improvements in treatment options, patients with advanced-stage NPC still have a poor prognosis. As early-stage NPC exhibits non-specific signs and symptoms, most patients present with an advanced stage of NPC at diagnosis. Recurrence and distant metastasis represent the leading causes of NPC-related death [2]. To date, the diagnosis and prognosis of NPC are determined mainly on the basis of the American Joint Committee on Cancer (AJCC) staging system [3]. However, patients with NPC differ in prognosis, even at the same stage [4]. Therefore, prognostic indicators with higher specificity and sensitivity remain to be discovered and applied to the diagnosis and individualized treatment of patients with NPC. In this context, there is an urgent need for new biomarkers to identify early-stage NPC patients at high risk of poor prognosis.

Accumulating evidence suggests that NPC is strongly associated with Epstein–Barr virus (EBV) infection [5]. EBV-DNA is currently recognized as a prognostic indicator of nasopharyngeal carcinoma [6, 7]. Many antibodies are expressed during the infection cycle of EBV; their use as prognostic markers for nasopharyngeal carcinoma remains controversial. The protein fragment d-dimer is an important prognostic marker for other tumors, but its role in NPC has not been well clarified.

The value of anti-EBV IgA antibodies (e.g., EA-IgA, VCA-IgA) and other EBV-related antibodies for NPC diagnosis has been reported [8]. Our previous work indicates that EBV-DNA and EBV-related antibodies have diagnostic value [9]. Further to our previous report, this meta-analysis was conducted to shed light on the prognostic value of these biomarkers in NPC patients through a literature search of published studies, data extraction, quality assessment and statistical processing.

## Methods

The data in this meta-analysis were presented strictly in accordance with the PRISMA reporting guidelines [10].

### Data sources and literature search strategy

Two independent investigators performed systematic searches of online databases, including PubMed, Embase, and Web of Science, for articles published up to February 1, 2021. The following search strategy was used: (nasopharyngeal and (cancer or tumor or carcinoma or neoplasm) and (EBV-DNA or ‘Epstein Barr virus’ or ‘capsid antigen-IgA’ or ‘early antigen antibody’ or ‘nuclear antigen antibody’ or ‘BRLF1 transcription activator IgG’ or EA-IgA or VCA-IgA or Rta-IgG or EBNA1-IgA or d-dimer) and (prognosis or survival or mortality)). The articles were first screened by title and abstract. Next, duplicate articles and those not published in English were eliminated. Finally, all extracted data from the selected articles were synthesized for analysis.

### Inclusion and exclusion criteria

The inclusion criteria were as follows: (1) the serum antibody levels of biomarkers were determined using enzyme-linked immunosorbent assay; (2) the recorded data included biomarkers and survival outcomes such as overall survival (OS), disease-free survival (DFS), progression-free survival (PFS), metastasis-free survival (MFS), local–regional failure survival (LRFS), relapse-free survival (RFS), and distant metastasis-free survival (DMFS). Studies published as case reports, conference abstracts, correspondence, or reviews were excluded.

### Data extraction and quality assessment

The data extracted from the articles included study characteristics (first author, publication year, country of origin, total number of patients, gender, and age); cut-off values of biomarkers, tumor–node–metastasis classification (TNM) stage, and treatment plan; survival outcomes (including OS, DFS, PFS, MFS, LRFS, RFS, and DMFS); and statistical evaluations, including hazard ratios (HR), 95% confidence intervals (95% CI), and *P*-values. The study quality was assessed independently by two investigators according to the Newcastle–Ottawa Scale (NOS) [11]. The NOS scores ranged from 0 to 9 (allocated as stars). Articles with six stars or more were evaluated as high quality. All dissenting opinions were discussed until a consensus was reached.

### Statistical analysis

*Review Manager (RevMan) [Computer program]. Version 5.4, The Cochrane Collaboration, 2020* was used for data analysis. HR and 95% CI were used to assess the prognostic values of independent biomarkers. The survival data were extracted by Engauge Digitizer 4.1 as Kaplan–Meier curves [12]. The inconsistency index (*I*^*2*^) was used to estimate the heterogeneity of individual studies, which facilitated generation of the pooled estimates. When heterogeneity across the studies was present (*I*^*2*^ > 50%), the random-effect model was selected; otherwise, the fixed-effect model was used. Sensitivity analysis was performed by deleting each included study in turn to explore the source of heterogeneity. The funnel plot, Begg’s bias test, and Egger’s test were applied to identify potential publication bias. *P* < 0.05 was considered statistically significant.

## Results

### Article search and study quality

**Fig. 1:**
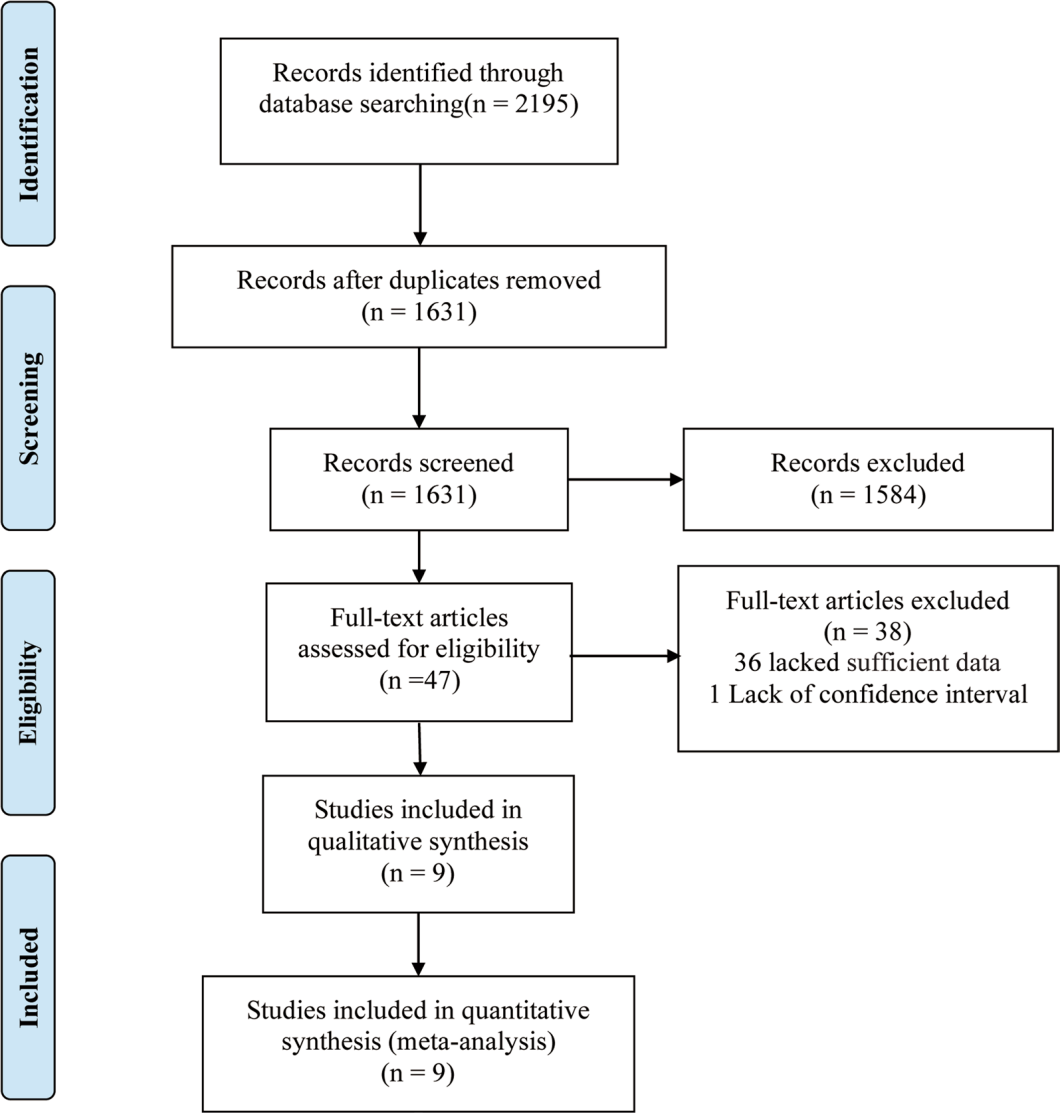
Flowchart of the study selection process

A total of 2195 studies were identified from Embase (n = 1077) and PubMed (n = 1118); 564 studies were excluded due to duplication. Nine studies, comprising 5729 patients with NPC, were eligible for this meta-analysis [13–22]. One study was rejected due to lack of confidence interval data [19]. Figure 1 shows a flowchart of the study selection process. Table 1 shows the major characteristics and detailed NOS scores of the included studies. All included studies were retrospective.

**Table 1:** Main characteristics of the nine studies included in the final analysis

Study	Country	Area	Patients	Gender		Mean age	TNM stage	Median follow-up time (month)	Markers	Survival outcomes	Quality score
				Male	Female						
He et al. [16]	China	Guangzhou	511	–	–	–	I–IV	45.2	d-Dimer, EBV-DNA	OS, DMFS	⭐⭐⭐⭐⭐⭐
Chen et al. [14]	China	Guangzhou	717	533	184	47	I–IV	31	d-Dimer, EBV-DNA, VCA-IgA, EA-IgA	OS, DMFS, DFS	⭐⭐⭐⭐⭐⭐⭐⭐
Xu et al. [21]	China	Guangzhou	703	539	164	–	I–IV	120	VCA-IgA, EA-IgA	OS	⭐⭐⭐⭐⭐⭐⭐
Feng et al. [15]	China	Guangzhou	143	118	24	49.6	I–IV	–	VCA-IgA, EA-IgA	OS	⭐⭐⭐⭐⭐⭐⭐⭐
Jin et al. [18]	China	Guangzhou	799	664	135	45.4	I–IV	15.4	VCA-IgA, EBV-DNA	OS	⭐⭐⭐⭐⭐⭐
Twu et al. [20]	China	Taiwan	114	87	27	46	I–IV	46	VCA-IgA, VCA-IgG, EBV-DNA	OS, RFS	⭐⭐⭐⭐⭐⭐
Cao et al. [13]	China	Guangzhou	198	156	42	44.5	I–IV	51.5	VCA-IgA, EA-IgA	OS	⭐⭐⭐⭐⭐⭐
Yao et al. [22]	China	Guangzhou	334	251	83	45	I–IV	–	VCA-IgA, EA-IgA	OS, DMFS, PFS, LRFS	⭐⭐⭐⭐⭐⭐
Jin et al. [17]	China	Xuzhou	164	117	47	–	I–IV	49.2	VCA-IgA, EA-IgA, EBV-DNA	OS, DMFS, DFS, LRFS	⭐⭐⭐⭐⭐⭐

*OS* overall survival, *PFS* progression-free survival, *DMFS* distant metastasis-free survival, *LRFS* local–regional failure survival, *MFS* metastasis-free survival, *DFS* disease-free survival, *RFS* relapse-free survival

### EA-IgA levels and survival outcome

**Fig. 2:**
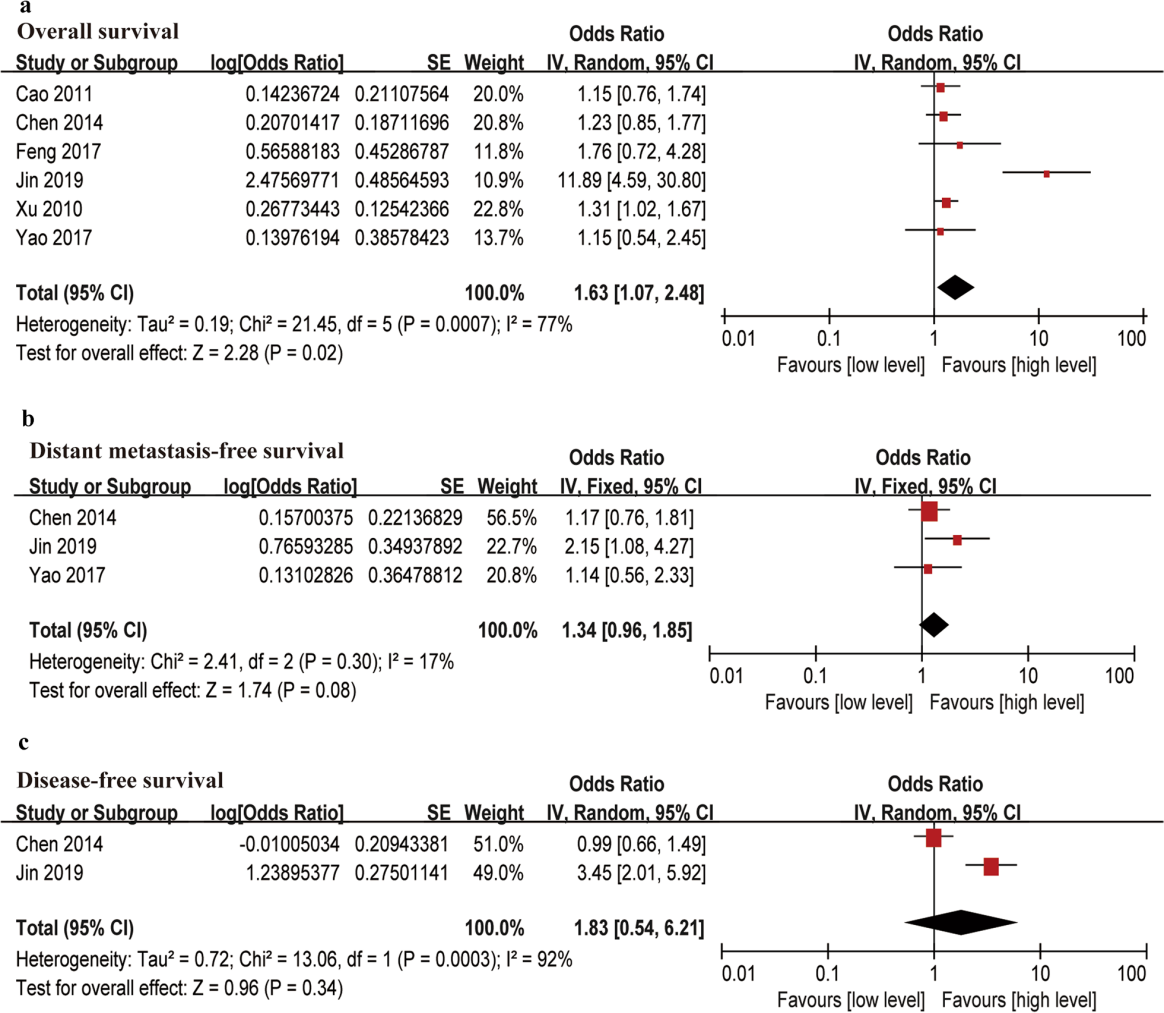
Forest plots of EA-IgA levels associated with survival outcomes. **a** Overall survival (OS), **b** Distant metastasis-free survival (DMFS), **c** Disease-free survival (DFS)

Six articles focused on the association between EA-IgA and OS. The meta-analysis found that high levels of EA-IgA predicted a poor prognosis with low OS (HR = 1.63, 95% CI 1.07–2.48) with the random-effect model (*I*^*2*^ > 50%). High levels of EA-IgA did not correlate with DMFS (HR = 1.34, 95% CI 0.96–1.85) or DFS (HR = 1.83, 95% CI 0.54–6.21) in patients with NPC (Fig. 2). Results of the meta-analysis also showed that patients with high EA-IgA levels had a greater probability of adverse survival outcomes than patients with low EA-IgA levels.

### VCA-IgA levels and survival outcome

**Fig. 3:**
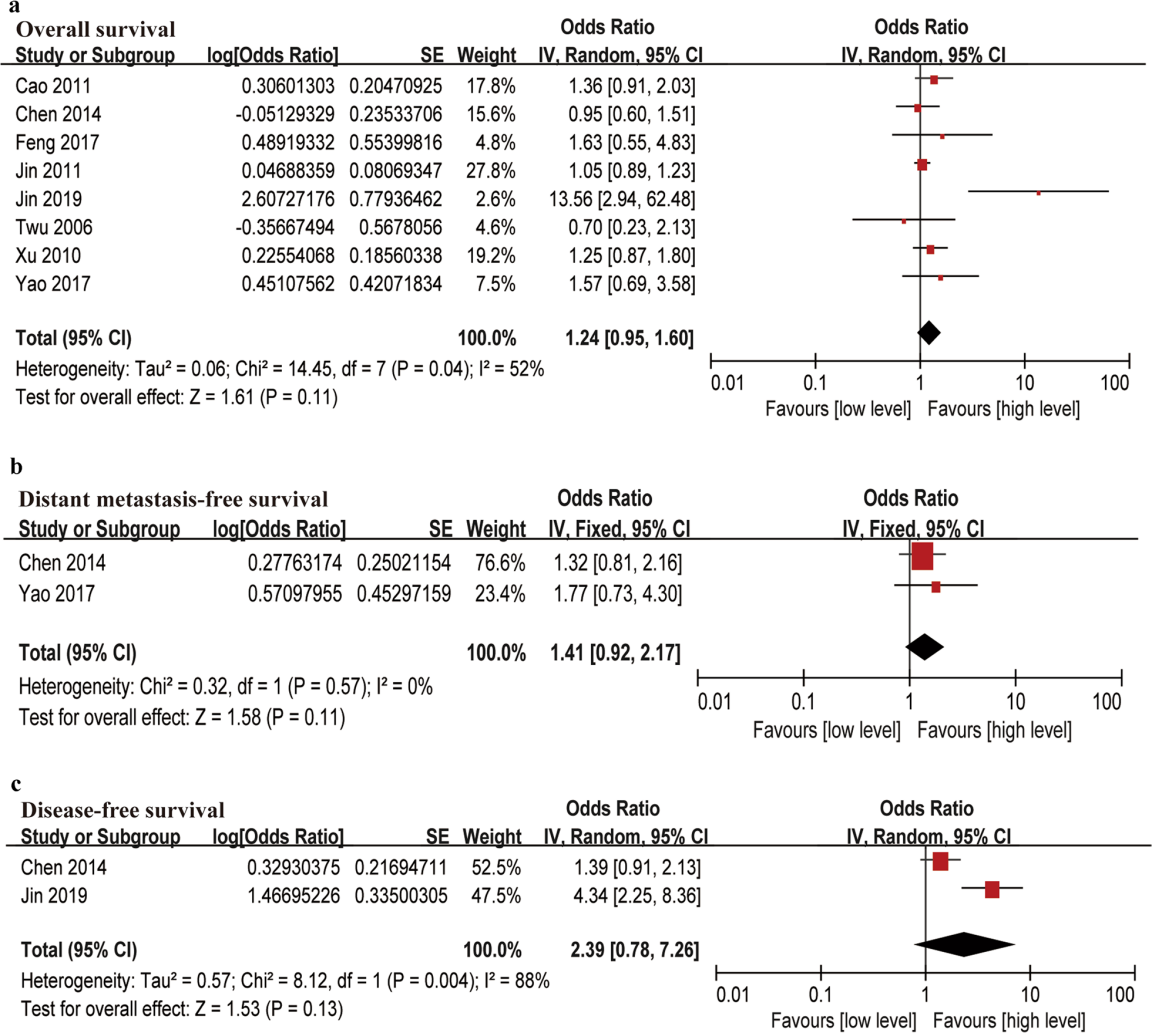
Forest plots of VCA-IgA levels associated with survival outcomes. **a** Overall survival (OS), **b** Distant metastasis-free survival (DMFS), **c** Disease-free survival (DFS)

Eight articles focused on the association between VCA-IgA and OS. The random-effect model was used for OS (*I*^*2*^ = 52%), and the fixed-effect model was used for DMFS and DFS (*I*^*2*^ ≤ 50%). The pooled meta-analysis indicated that high levels of VCA-IgA were not associated with OS (HR = 1.24, 95% CI 0.95–1.60), DMFS (HR = 1.41, 95% CI 0.92–2.17) or DFS (HR = 2.39, 95% CI 0.78–7.26) in patients with NPC (Fig. 3).

### d-Dimer levels and survival outcome

**Fig. 4:**
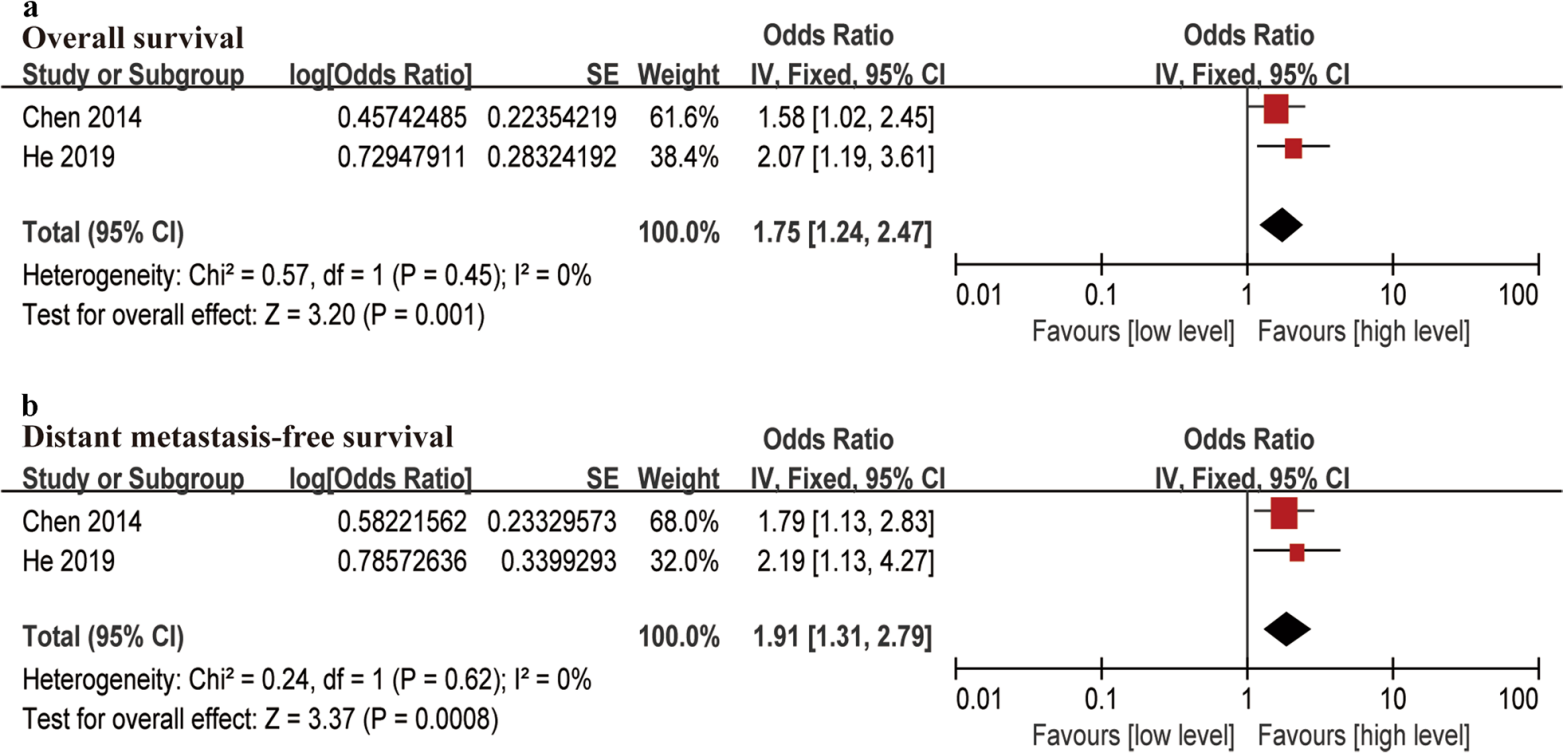
Forest plots of d-Dimer levels associated with survival outcomes. **a** Overall survival (OS), **b** Distant metastasis-free survival (DMFS)

Two articles focused on the association between d-dimer and OS. By using the fixed-effect model (*I*^*2*^ ≤ 50%), the pooled meta-analysis indicated that high levels of d-dimer in NPC patients predicted a poor prognosis with low OS (HR = 1.75, 95% CI 1.24–2.47) and low DMFS (HR = 1.91, 95% CI 1.31–2.79) (Fig. 4). Patients with high d-dimer levels had a greater probability of poor survival outcomes.

### Sensitivity analysis and publication bias

Sensitivity analysis suggested that the results of EA-IgA levels and OS were influenced by data from Jin et al. [17]. After the data from this article were removed, the heterogeneity reduced to *I*^*2*^ < 50%. By using the fixed-effect model, we obtained consistent results: high levels of EA-IgA in NPC patients predicted a poor prognosis with low OS (HR = 1.75, 95% CI 1.24–2.47).

**Fig. 5:**
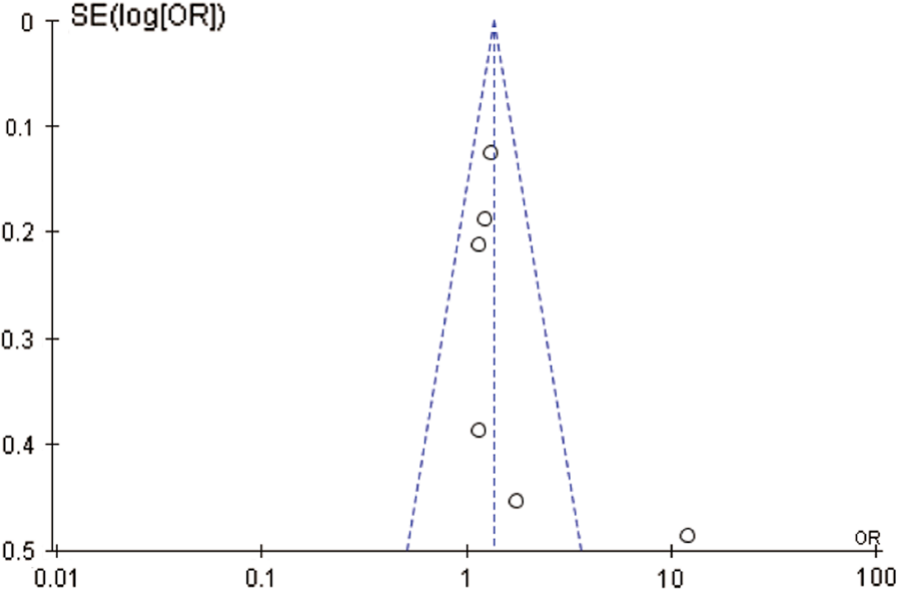
Funnel plot

The funnel plot and Egger’s test were used to investigate potential publication bias (Fig. 5). Using Egger’s test, we found no evidence of bias in the meta-analysis that would otherwise explain the observed association between EA-IgA levels and OS (*P* = 0.365). *P* > 0.05 indicates no significant publication bias.

## Discussion

The determination of plasma EBV-DNA levels is widely used to diagnose NPC and evaluate therapeutic efficacy [23, 24]. The present study suggests that EBV-DNA levels could be used to predict the prognosis of NPC. The meta-analysis comprising nine studies with 5729 patients also explore the significance of other clinical indicators for predicting NPC prognosis. Our findings demonstrate that the levels of EA-IgA and d-dimer, but not VCA-IgA, are associated with survival endpoints.

Aggregate results reveal that EA-IgA can be used as an indicator of OS in patients with NPC. VCA-IgA does not seem to be an indicator of prognosis in patients with NPC. d-Dimer may be a good indicator to predict OS and DMFS in patients with NPC. However, few studies have investigated d-dimer, so there is an urgent need to test more patient samples for d-dimer to improve the evidence level of this biomarker. Furthermore, several studies indicated that biomarkers such as Rta-IgG, Zta-IgA, and EBNA1-IgA may also be prognostic indicators of NPC. Our meta-analysis did not assess these biomarkers due to the limited number of patients included in published prognostic studies.

Recent studies in NPC patients have identified antibodies against EBV antigens, including early antigen (EA), viral capsid antigen (VCA) and Epstein–Barr virus nuclear antigen 1 (EBNA1) [25]. When EBV enters the lytic cycle, Rta and Zta are encoded and then EA is encoded [26]. At the end of the EBV proliferation cycle, VCA is expressed. During the cycle of EBV proliferation, expression of these antigens induces a strong antibody response in patients with NPC [27]. The detection of EBV infection by measuring these antibody expression levels allows diagnosis and prognosis prediction of NPC. These antibodies may be detected at any period in clinical practice. Therefore, detection of these antibodies can improve the accuracy of prediction and guide clinicians to quickly adjust their treatment plans.

In recent years, the fibrinolytic system has attracted attention as a possible regulator of cancer progression [28, 29]. Plasma d-dimer has been used to evaluate the prognosis of various cancers, such as NPC, small-cell lung cancer [30] and gastrointestinal cancer [31]. The survival outcomes related to d-dimer in non-NPC cancers are similar to our meta-analysis results.

Prediction of NPC prognosis should be based on the patient's condition. Timely adjustments of treatment plans are expected to improve the prognosis and prolong the survival time of patients with NPC. Data from multiple antibody assays can improve prediction accuracy. Therefore, future research should aim to improve the combination of biomarkers used for prognosis prediction in patients with NPC.

Previously, EBV-DNA and other blood markers (such as LMP1) have been mainly used individually as prognostic indicators of NPC [32, 33], which lacks accuracy. We speculate that combining multiple biomarkers can improve the accuracy of prognosis predictions, but this requires validation in future studies. Because most research has previously focused on EBV-DNA, our present study investigated the prognostic values of other indicators for NPC. Our previous study reported that EA-IgA can be used for the diagnosis of NPC [9]. Combined with data from this meta-analysis, we show that these biomarkers can be used as both diagnostic and prognostic indicators, because these indicators can be detected during the EBV infection cycle. This illustrates the importance of testing these indicators at different disease stages.

There are some limitations to this study. First, all analyses were based on data extracted from nine published studies—the number of studies included in the final analysis was low, and all these studies were from China, which may increase the risk of selection and publication bias. Second, all nine studies were retrospective, which may increase heterogeneity. Third, expanding the analysis to other important antibodies expressed in response to the EBV infection cycle would be beneficial, although current data are limited. Finally, differences in follow-up duration and treatment regimens across the nine studies may also influence the results. A larger, prospective, randomized, multicenter clinical trial is required to confirm the prognostic potential of EA-IgA, d-dimer and VCA-IgA in NPC.

## Conclusions

In conclusion, EA-IgA and d-dimer are important prognostic predictors in NPC. Combined detection of multiple indicators should improve the accuracy of prediction. Regular monitoring of relevant prognostic indicators will help clinicians make timely changes in treatment regimens and will improve patient survival. This research will inspire future prospective studies to validate whether panel detection of EA-IgA, d-dimer and EBV-DNA could more accurately predict the prognosis of NPC patients.

## Data Availability

The datasets used during the current study are available from the corresponding author on reasonable request.

## Abbreviations

NPC: Nasopharyngeal carcinoma
EBV: Epstein–Barr virus
EA: Early antigen
VCA: Viral capsid antigen
EBNA1: Epstein–Barr virus nuclear antigen 1
TNM: Tumor–node–metastasis classification
CI: Confidence interval
*I*^*2*^: Inconsistency index
HR: Hazard ratios
OS: Overall survival
DFS: Disease-free survival
PFS: Progression-free survival
MFS: Metastasis-free survival
LRFS: Local–regional failure survival
RFS: Relapse-free survival
DMFS: Distant metastasis-free survival
NOS: Newcastle–Ottawa Scale
